# Dupilumab-Related Diabetes Mellitus With Reversal of Symptoms in a Non-genetically Predisposed Patient

**DOI:** 10.7759/cureus.53080

**Published:** 2024-01-27

**Authors:** Tanner C Blaylock, Daniel Leon

**Affiliations:** 1 College of Allopathic Medicine, Nova Southeastern University Dr. Kiran C. Patel College of Allopathic Medicine, Fort Lauderdale, USA; 2 Internal Medicine, Chen Senior Medical Center, Plantation, USA

**Keywords:** monoclonal antibodies (mabs), immunogenicity, autoimmune type 1 diabetes mellitus, autoimmune disease, dupilumab

## Abstract

Dupilumab is a fully humanized monoclonal antibody that binds to IL-4 receptors and blocks IL-4 and IL-13 mediated T-helper 2 (Th2) responses. Dupilumab is estimated to be used by over 600,000 patients worldwide for the treatment of atopic dermatitis and other immunologic conditions. Recently, a 66-year-old male patient being treated for atopic dermatitis with dupilumab presented to the clinic with complaints of polyuria and polydipsia. Upon initial testing, the patient was found to have considerable hyperglycemia. Upon genetic testing, he showed no predisposition for autoimmune diabetes and was negative for type I diabetes mellitus-associated human leukocyte antigen (HLA) genes. After immediate cessation of dupilumab, and with subsequent insulin therapy, the patient was able to obtain glycemic control. Following taper and eventual cessation of insulin therapy and over the course of seven months, the patient was able to achieve a full resolution of symptoms and his glycosylated hemoglobin (HgBA1c) levels returned to normal ranges. This case represents only the second documented case of dupilumab-induced diabetes mellitus and is the first known documented case of dupilumab-induced diabetes mellitus in a non-genetically predisposed individual. This case also describes a previously unobserved spontaneous resolution of symptoms upon cessation of the drug. This case further illustrates the potential existence of immunogenic or immunomodulatory side effects of the monoclonal antibody dupilumab that can affect patients who are both genetically and non-genetically predisposed to autoimmune diabetes mellitus.

## Introduction

Fully humanized monoclonal antibodies have been used with increased frequency to treat many conditions ranging from autoimmune disorders to cancer. Although fully humanized monoclonal antibodies have been uniquely engineered to reduce immunogenic effects, such unwanted consequences can still exist. Despite being fully humanized, monoclonal antibodies, when administered frequently over long periods, are still capable of producing anti-drug antibodies (ADAs). These ADAs can lead to adverse effects such as decreased efficacy of the drug, triggering of autoimmune reactions, or cross-reactivity with endogenous hormones such as erythropoietin (EPO) or growth hormone (GH) [1,2].

Likewise, dupilumab is a humanized monoclonal antibody that acts to block IL-4 receptors at the alpha-subunit [3]. This functions to diminish the effects of both IL-4 and IL-13, which play a role in the T-helper 2 (Th2) response. Previously, Kurokawa et al. described a case depicting dupilumab-related diabetes in a young female patient who was later determined to be predisposed to type 1 diabetes mellitus due to the presence of known type 1 diabetes mellitus (T1DM)-associated human leukocyte antigen (HLA) genes: human leukocyte antigen class II histocompatibility, D-related beta chain (HLA DRB1) (specifically, D related beta chains 3 and 4 (DR3, DR4)) and human leukocyte antigen class II histocompatibility, DQ-related beta chain (HLA DQB1) (specifically the DQ-related beta chain 8 (DQ8)). In that case, it was theorized that dupilumab was acting to reduce the Th2 response in the already genetically susceptible patient leading to increased T-helper 1 (Th1) related activity and induction of diabetes mellitus type 1 [4].

Presently, we describe the case of a patient who presented with signs and symptoms of diabetes mellitus but who, upon further testing, was not found to have T1DM-associated HLA genes or other genetic predispositions. Additionally, the patient, after cessation of dupilumab, returned to normal blood glucose and glycosylated hemoglobin (HgA1c) levels and was completely tapered off pharmacological intervention including insulin therapy without recurrence of the disease. This case builds upon the knowledge of previously described dupilumab-related diabetes while widening the potential susceptible patient population beyond individuals with T1DM-associated HLA genes. Furthermore, this case shows the potential for reversibility of the described dupilumab-related diabetes upon cessation of the drug in select patient populations.

## Case presentation

A 66-year-old man was being treated for refractory atopic dermatitis, affecting the palmar surfaces of the hands bilaterally, with dupilumab and had obtained clinical remission of symptoms with treatment. In June of 2022, the patient was started with 600 mg administered as two 300 mg/2mL loading doses and continued with 200 mg/1.4mL injection pen of Dupixent® (dupilumab), which was injected subcutaneously every two weeks for maintenance, which he took for seven months.

In addition to atopic dermatitis, the patient had been previously diagnosed with hypertension, benign prostatic hyperplasia, and mild chronic obstructive pulmonary disease (COPD), for which he was taking daily medications, amlodipine-olmesartan 5-20 mg, tamsulosin 0.4 mg, and Spiriva (tiotropium bromide) 18 mcg inhaler, respectively. Prior to the start of dupilumab, the patient had been screened for type II diabetes mellitus every three years, per the United States Preventative Services Task Force screening guidelines [5], with no evidence of type II diabetes mellitus. The patient's most recent HbA1c prior to initiation of therapy with dupilumab was 5.4% (normal <5.7%) in April 2022.

In March of 2023, after roughly 14 injections of dupilumab, the patient presented to the clinic for a routine health appointment. On arrival, his vital signs were within normal limits. His height was 63 inches, weight was 152 pounds, and BMI was 26.9. Upon review of symptoms, the patient reported polyuria as his only symptom. During this visit, his fasting blood glucose level was obtained and was found to be 436 mg/dL. Given that there was no history of diabetes or prediabetes (the patient’s previous HbA1c tested in April of 2022 was 5.4%), the patient’s blood was subsequently redrawn and sent to the lab for repeat verification. The results of the repeat labs were a fasting glucose level of 641 mg/dL with an HbA1c of 12.4%, sodium of 130 nmol/L (reference range: 134-144 nmol/L), potassium of 6.3 nmol/L (reference range: 3.5-5.2 nmol/L), and chloride of 93 nmol/L (reference range: 96-106 nmol/L).

Upon confirmation of lab reports, diabetes mellitus (unknown etiology) with hyperglycemia was diagnosed, and therapy was begun. The patient was initially treated with a 1-liter bolus of IV 0.9% normal saline followed by seven units of IV insulin lispro. This was followed by subcutaneous injection of seven units of insulin lispro with another 1-liter bolus of IV 0.9% normal saline being administered. The patient was instructed to discontinue the dupilumab medication and to return to the office the next day for subsequent insulin treatment and follow-up education.

Upon follow-up visits, the patient was started on Insulin lispro (75/25) subcutaneous injection given twice a day, with 15 units given in the morning (AM) and 20 units given in the evening (PM). At a subsequent May 2023 visit, the insulin dose was titrated to 24 units in the morning (AM) and 20 units in the evening (PM), and glycemic control was achieved. By a follow-up visit in June 2023, the patient had reported glycemic control (office non-fasting glucose check 138 mg/dL) with the use of 10 units of insulin in the morning (AM) and 10 units in the evening (PM). After subsequent follow-up visits and by September of 2023, the patient was able to obtain a HgA1c level within normal limits (<5.7%) without the use of insulin and without significant reported lifestyle modifications, corresponding to the timeframe in which the patient was taken off dupilumab therapy (Table 1). Labs were drawn to assess genetic predispositions and the outcomes were as seen in Table 2.

**Table 1:** Complete metabolic panel with hemoglobin A1c levels BUN: blood urea nitrogen; eGFR: estimated glomerular filtration rate; Cr: creatinine; hemoglobin A1c: glycosylated hemoglobin

Test	Results	Previous results		Reference range	Units
	April 6, 2023	March 29, 2023	April 1, 2022		
Glucose	641	436	not obtained	70-100	mg/dL
BUN	19	17	not obtained	6-24	mg/dL
Creatinine	1.86	1.66	not obtained	.7-1.3	mg/dL
eGFR	39	45	not obtained	>90	mL/min/1.73
BUN/Cr Ratio	10	10	not obtained	10-20	N/A
Sodium	130	133	not obtained	134-144	nmol/L
Potassium	6.3	5.3	not obtained	3.5-5.2	nmol/L
Chloride	93	93	not obtained	96-106	nmol/L
Hemoglobin A1c	12.4	not obtained	5.4	<5.7	%

**Table 2:** HLA genetic testing and diabetes autoantibody panel HLA: human leukocyte antigen

Test	Result - August 9, 2023	Allele/reference range	Units
HLA-A	A * 23: 01: 01G	N/A	N/A
HLA DQB1 (DQ)	DQB1 * 02: 02: 01 :01	First allele	N/A
	DQB1 * 03: 19: 01 :01	Second allele	N/A
HLA DRB1 (DR)	DRB1 * 07: 01 :01G	First allele	N/A
	DRB1 * 11: 01: 02G	Second Allele	N/A
IA-2 autoantibodies	<7.5	<7.5 = negative	U/mL
ZNT8 antibodies	<15	<15= negative	U/mL
C-peptide	3.2	1.1-4.4	ng/mL
GAD-65 autoantibody	<5.0	0.0-5.0	U/mL
Antipancreatic islet cells	Negative	Negative: < 1:1	U/mL

## Discussion

In recent years, fully humanized monoclonal antibody therapy has dominated pharmaceuticals as a treatment option for autoimmune diseases with less potential immunogenic side effects. Dupilumab has been marketed as a fully humanized monoclonal antibody against the IL-4 receptor, allowing it to mitigate the effects of both IL-4 and IL-13, thus dampening the Th2 helper T-cell response of the immune system [3]. In a recent case report, Kurokawa et. al. reported a case of dupilumab-induced diabetes mellitus in a young female. In that case report, it was theorized that an imbalance of Th2- and Th1-mediated immunity due to the ability of dupilumab to reduce the body’s Th2 response, could have been an underlying mechanism for the induced diabetic response observed in their case [4]. However, the patient described in the aforementioned study was shown to have a predisposition to type 1 diabetes due to the presence of known T1DM-associated HLA genes (DR3, DR4, and DQ8). In the present case, sequencing of T1DM-associated HLA genes yielded no such results. Upon testing, the patient’s HLA gene results showed the presence of genes not known in the present literature to carry an association with type-1 diabetes (HLA DR7, DR11, DQ2, DQ3) [6]. The patient had negative titers for common antibodies found in autoimmune diabetes and his C-peptide levels were within normal limits. Furthermore, our patient, upon cessation of dupilumab, eventually returned to normal HgA1c levels without the need for insulin therapy. These variations in case presentation and disease course naturally lead to the notion that other pathogenetic processes of dupilumab-related diabetes mellitus must plausibly exist for non-genetically predisposed individuals to experience diabetes mellitus symptoms following long-term dupilumab use.

ADAs are a known immunogenic consequence of monoclonal antibody therapy. ADAs have the ability to not only affect the pharmacokinetics and bioavailability of therapeutic drugs but can, in severe cases, lead to immune complex disease, allergic reactions, and autoimmune reactions [7]. These ADAs can be either binding antibodies (BAbs), which primarily work to either increase the clearance of the drug or sustain its half-life, or neutralizing antibodies (NAbs). NAbs, which typically bind to the functional drug epitope site, have been linked to the ability to cause conditions such as pure red blood cell aplasia and thrombocytopenia due to their ability to bind off target epitopes including endogenous hormone [8-10]. In case reports described by Casadevall et. al. and Li et. al., researchers observed that chronic administration of recombinant hormone (erythropoietin (EPO) and thrombopoietin, respectively) led to the development of pure red cell aplasia and thrombocytopenia due to suspected ADA development and subsequent cross-reactivity and inactivation of endogenous hormone [9,10]. Although the therapy given in these cases was recombinant hormone, it is not out of the question that a similar mechanism could be at play in the patient presented in our case via the binding of NAbs formed against dupilumab to endogenous hormones such as insulin causing a decreased effect of endogenous insulin and induction of diabetes mellitus symptoms.

Other cases reported in the literature have shown examples of monoclonal antibody therapy resulting in the reduction of endogenous hormone levels, such as EPO. In one such case, prolonged atezolizumab therapy led to reduced EPO levels and subsequent drug-induced pure red blood cell aplasia in a 45-year-old patient [11]. Other studies showed that common cancer-fighting program death 1 (PD-1) and program death ligand 1 (PDL-1) inhibitors have been shown to have numerous autoimmune endocrine side effects such as drug-induced T1DM [12,13]. Pembrolizumab, a humanized monoclonal antibody against PD-1 used in the treatment of common cancers such as melanoma, lung cancer, and Hodgkin lymphoma, is one such example of a drug that was implicated in the induction of T1DM [14]. In these instances, the prescribed theory attributes the induction of autoimmune destruction of endogenous hormones to the drugs’ capability to bind off-target tissue or hormone epitopes, which produces the undesired side effect [15]. These examples provide a framework for yet another plausible explanation for the phenomenon observed in the patient presented in this case.

Based on the review provided above and owing to the observed case present in this report, we postulate that dupilumab-related diabetes is a potential concern for more than T1DM genetically predisposed individuals and that further investigation into the pathogenicity of dupilumab is warranted along with investigative studies into the pathogenicity of the biologics class of therapeutics.

## Conclusions

To our knowledge, this is the first report of dupilumab-related diabetes in a non-genetically predisposed individual who also obtained full reversal of symptoms upon cessation of dupilumab. The most recent literature has reported the ability of dupilumab therapy to induce diabetes symptoms in an individual with T1DM-associated HLA genes. No such literature, however, has addressed the risk of dupilumab-related diabetes in non-genetically predisposed individuals. As such, our case brings to light the need for further characterization of the pathogenicity of dupilumab. We believe that the undertaking of further research into the pathology of anti-drug antibodies or other plausible pathogenic theories is warranted. Elucidating the exact pathogenicity of dupilumab-related diabetes is important as this case demonstrates that the general population may be susceptible to the described side effects.
